# Adverse Obstetric Outcomes in Pregnant Women Using Natalizumab for the Treatment of Multiple Sclerosis: A Systematic Review

**DOI:** 10.7759/cureus.29952

**Published:** 2022-10-05

**Authors:** Vaiishnavi Ramesh, Collins O Opara, Farhana Yaqoob Khan, Gargi Kabiraj, Humaira Kauser, Jaimee J Palakeel, Mazin Ali, Phani Chaduvula, Sanika Chhabra, Smriti Lamsal Lamichhane, Safeera Khan

**Affiliations:** 1 Family Medicine, California Institute of Behavioral Neurosciences & Psychology, Fairfield, USA; 2 Radiation Medicine, California Institute of Behavioral Neurosciences & Psychology, Fairfield, USA; 3 Pathology, California Institute of Behavioral Neurosciences & Psychology, Fairfield, USA; 4 Medicine, California Institute of Behavioral Neurosciences & Psychology, Fairfield, USA; 5 Medical School, California Institute of Behavioral Neurosciences & Psychology, Fairfield, USA; 6 Neurology, California Institute of Behavioral Neurosciences & Psychology, Fairfield, USA; 7 Telegeriatrics, Michigan State University, Grand Rapids, USA; 8 General Medicine, California Institute of Behavioral Neurosciences & Psychology, Fairfield, USA; 9 Internal Medicine, California Institute of Behavioral Neurosciences & Psychology, Fairfield, USA

**Keywords:** treatment, disease-modifying therapy, pregnancy, natalizumab, multiple sclerosis

## Abstract

Multiple sclerosis is a common disease in women of childbearing age, characterized by demyelination of the central nervous system. Among the different treatment options available, disease-modifying therapies (DMTs) are the most efficacious, and natalizumab (NAT) is an injectable DMT best for relapsing-remitting multiple sclerosis. However, it comes under pregnancy category C drug classification. This systematic review aims to analyze the adverse outcomes of using NAT during pregnancy.

PubMed/Medline, PubMed Central (PMC), ScienceDirect, and Google Scholar were the databases used to search for articles. Appropriate keywords and Medical Subject Headings (MeSH) strategy were used to identify relevant articles. Articles were then screened using inclusion/exclusion criteria followed by the title and abstract screening. The Joanna Briggs Institute (JBI) quality appraisal tools were used for quality check, and nine articles were finalized for review.

NAT suspension during pregnancy is shown to have a high risk of disease relapse. Despite the risk of mild hematological abnormalities in the newborn and the risk of spontaneous abortions at the same rate as that of the general population, NAT use can be considered safe in pregnancy. These adverse outcomes can be minimized by strict monitoring of patients. Studies of better quality with larger sample sizes are needed for further investigation.

## Introduction and background

Multiple sclerosis is an autoimmune disease of the central nervous system with a prevalence of one out of 1000 people in a healthy population [1]. It is characterized by demyelination and neurodegeneration due to inflammatory plaque formation in the brain and spinal cord [2]. Women of childbearing age are more commonly affected, the median age of disease onset being approximately 30 years [3]. Although various treatment options are available, disease-modifying therapies (DMTs) have the highest efficacy for treating active multiple sclerosis. On account of this, more importance is being given to the safety of using DMTs for treating multiple sclerosis, especially during pregnancy [3].

Natalizumab (NAT), a monoclonal antibody, is one such DMT that reduces inflammation in the central nervous system by blocking very late antigen type 4 (VLA-4), usually located on the surface of lymphocytes. It has been approved to treat active relapsing-remitting multiple sclerosis (RRMS) [3].

Previous systematic reviews and studies have failed to provide adequate information about the consequences of DMT use in pregnancy [4]. NAT is classified as a category C drug in pregnancy, suggesting that there are not enough well-controlled studies in humans [3,4]. No teratogenic effects with NAT use have been found in animal studies, albeit some results of increased abortion rates have been reported [3].

In addition, NAT increases the risk of opportunistic infections by weakening the immune systems of some patients taking it [5]. Maternal antibodies, including monoclonal antibodies given for therapeutic purposes, can cross the placental barrier during the second trimester of pregnancy, and their transport gradually increases with increasing gestational age. As a result, studies have shown a risk of hematological disorders such as anemia and thrombocytopenia in the newborns of mothers exposed to NAT during their third trimester [6]. However, these hematological alterations were back to normal within four months after birth in most patients, without much intervention [7].

Although NAT is a highly efficacious drug for patients with RRMS, studies have shown a relapse of the disease when its usage is interrupted, some as early as four weeks. Therefore, caution is needed to balance the benefits and risks of NAT exposure during pregnancy [8]. This is done individually to avoid rebound disease symptoms with its suspension. Since there is some evidence suggesting that NAT usage is associated with certain complications in pregnancy, this needs to be studied in detail to decide if NAT can be safely continued post conception. 

We conducted a systematic review to analyze the overall adverse obstetric outcomes in patients who have continued to use NAT for their multiple sclerosis treatment during pregnancy.

## Review

Methods

This systematic review adhered to the Preferred Items for Systematic Reviews and Meta-Analyses (PRISMA) 2020 guidelines [9].

Search Strategy and Databases

PubMed/ Medline, PubMed Central (PMC), ScienceDirect, and Google Scholar were used to identify full-text relevant published articles. Appropriate keywords that are relevant to the topic and Medical Subject Headings (MeSH) terms were used to systematically find all the available papers. The search strategy also included Booleans "AND" and "OR". The MeSH strategy used was: ("Multiple sclerosis/drug therapy"[MeSH Terms] OR "Multiple sclerosis/therapy"[MeSH Terms]) AND ("Natalizumab/adverse effects"[MeSH Terms] OR "Natalizumab/pharmacology"[MeSH Terms] OR "Natalizumab/toxicity"[MeSH Terms]) AND "Pregnancy"[MeSH Terms].

Keywords for other databases included pregnancy, natalizumab, multiple sclerosis, and disease-modifying therapy.

After identifying relevant articles, the corresponding authors worked independently to screen further these papers based on their titles, abstracts, and eligibility criteria. No automation tools were used in this process.

Inclusion and Exclusion Criteria

Articles published in the last ten years were included starting from October 2011 to November 2021, with the main focus on the pregnant population. Only articles published in English were selected. Observational studies, case reports, reviews, meta-analyses, and systematic reviews were included. Any grey literature, articles focusing on the non-pregnant population, and animal studies were excluded.

Quality Check and Risk of Bias Assessment

A total of 24 articles were considered for quality check, which included 18 observational studies, one case report, and one case series. All the observational studies, the case report, and the case series were screened using Joanna Briggs Institute (JBI) quality appraisal tools. Only those articles that fulfilled >65% of the quality checklist criteria were included in the review. Therefore, the risk of bias was low.

Results

Study Identification

**Figure 1:**
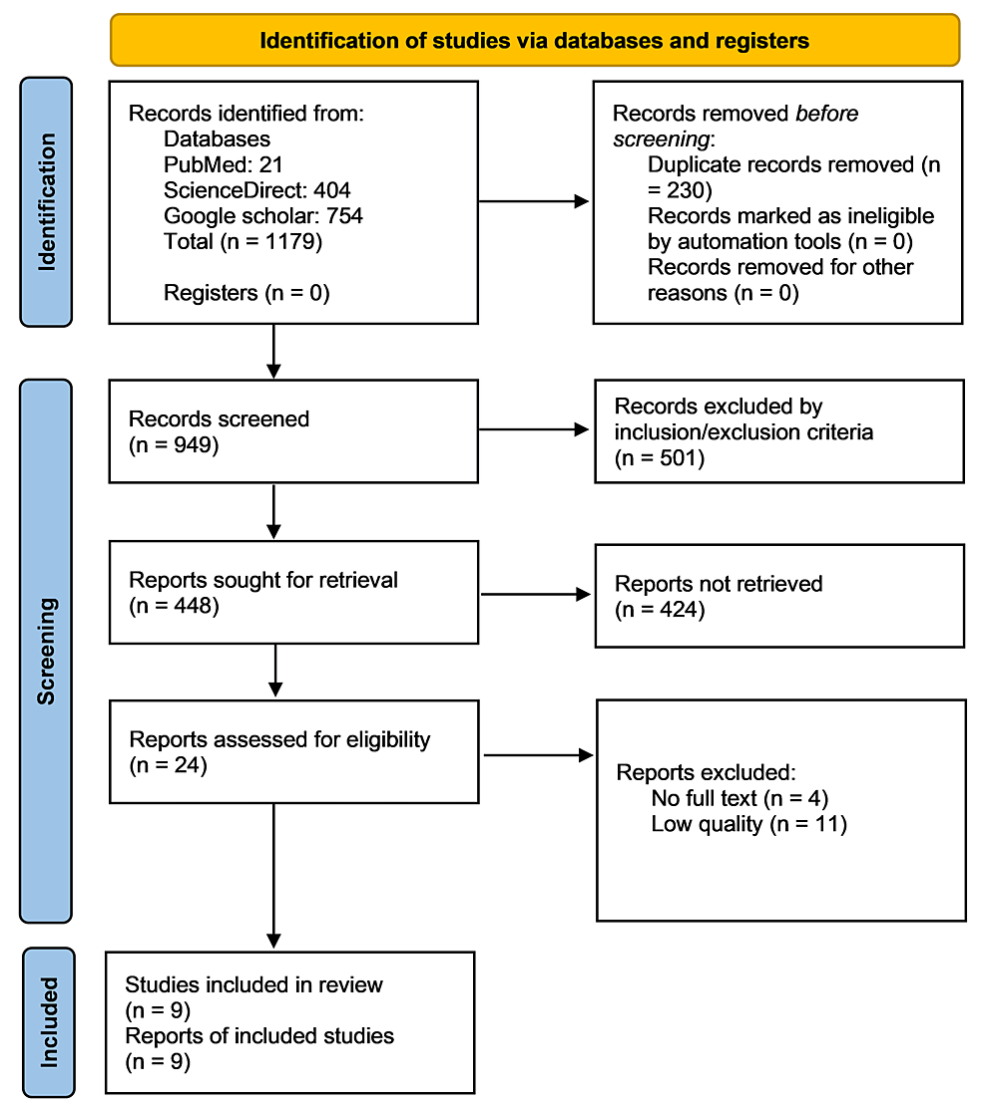
PRISMA flow diagram PRISMA= Preferred Reporting Items for Systematic Reviews and Meta-Analyses Source: [9]

Using different search strategies, 1,179 articles were identified: 21 from PubMed, 404 from ScienceDirect, and 754 from Google Scholar. Out of these, 230 duplicate articles were removed. The remaining 949 articles were filtered based on the inclusion/exclusion criteria, of which 448 articles were included and 501 excluded. These 448 articles were further screened based on their titles and abstracts, and irrelevant articles were removed, leaving behind 24 articles. Upon checking for full-text availability, four articles were removed due to the unavailability of the full text. Of the remaining 20 articles, nine fulfilled the quality assessment criteria and were finalized. The nine articles included seven observational (cohort) studies, one case report, and one case series. This entire process of study identification is depicted in the Preferred Reporting Items for Systematic Reviews and Meta-Analyses (PRISMA) flow diagram shown in Figure 1.

A brief description of the studies included in this systematic review is shown in Table 1.

**Table 1:** Characteristics of studies included in this systematic review MS= Multiple sclerosis; DMT= Disease modifying therapy; H-DMT= Highly effective Disease modifying treatment; EDSS= Expanded disability status scale; NAT= Natalizumab; MAbs= Monoclonal antibodies; RRMS= Relapsing remitting multiple sclerosis; DM and HC= Disease-matched and Healthy control; MACDP= Metropolitan Atlanta Congenital Defects Program; SA= Spontaneous abortions; CA= Congenital anomalies p <0.001= calculated probability. Indicates that there is less than one in a thousand chance that the study is insignificant.

Author	Year	Study Design	Purpose	Result
Proschmann et al. [3]	2018	Cohort study	To evaluate the transfer of NAT into breastmilk and into the serum of newborns whose mothers continued to use NAT during pregnancy.	NAT can cross the placental barrier before delivery and into breast milk. Neonates may have a risk of hematological abnormalities.
Godano et al. [6]	2021	Case report	To evaluate a case of documented anemia in a newborn whose mother with RRMS was treated with NAT throughout pregnancy.	NAT can cause disorders of hematopoiesis in newborns of patients treated during pregnancy.
Ebrahimi et al. [8]	2015	Cohort study	To compare the pregnancy outcomes in women with RRMS exposed to NAT in early pregnancy, to DM and HC groups of women.	Exposure to NAT does not appear to increase the risk of adverse pregnancy outcomes in comparison to a DM group not exposed to NAT.
Bsteh et al. [10]	2020	Cohort study	To examine the reciprocal effects of pregnancy, MS, and DMTs.	Use of H-DMT was associated with a higher risk of relapse and EDSS progression.
Portaccio et al. [11]	2018	Cohort study	To assess the risk of disease reactivation after NAT suspension, compared to patients who were untreated or received other injectable agents.	The relapse rate was higher during and after pregnancy in women treated with NAT (p < 0.001).
Ciplea et al. [12]	2020	Cohort study	To assess possible adverse effects on breastfed infants if mothers are exposed to MAbs during and after pregnancy.	NAT exposure was associated with lower birth weight and more hospitalizations in the first year of life. No negative impact on overall infant health was observed.
Haghikia et al. [13]	2014	Case series	To evaluate the hematological and birth outcomes of 13 infants born to 12 mothers exposed to NAT during the third trimester of pregnancy.	Mild anemia and thrombocytopenia were observed in 10 out of 13 infants.
Portaccio et al. [14]	2018	Cohort study	To assess fetal risks (specifically SA and CA) associated with NAT exposure in women during pregnancy.	Increased risk of SA was seen with exposure during the first trimester but within the limits expected in the general population.
Friend et al. [15]	2016	Cohort study	To evaluate the pregnancy outcomes of women with MS exposed to NAT within three months before or during pregnancy.	The overall rate of major birth defects was higher than that observed by MACDP, but no specific pattern suggestive of a drug effect was observed.

Discussion

The introduction of NAT as a treatment option for multiple sclerosis led to new challenges for neurologists and patients planning for pregnancy. On average, a minimum of 20% to 30% of women diagnosed with multiple sclerosis decide to have children [10]. Preclinical studies recommended a three-month washout before conception and avoiding NAT during pregnancy. However, close to one-third of the patients had reported disease reactivation within two to six months of NAT suspension [11].

Subsequently, the decision to continue NAT during pregnancy was made on an individual patient-to-patient basis. Some adverse events have been reported in those patients who are continuing treatment, including spontaneous abortions and hematological abnormalities in neonates as the most common.

This systematic review analyzes the adverse outcomes of pregnant patients using NAT, along with the effects of stopping NAT treatment before pregnancy.

**Figure 2:**
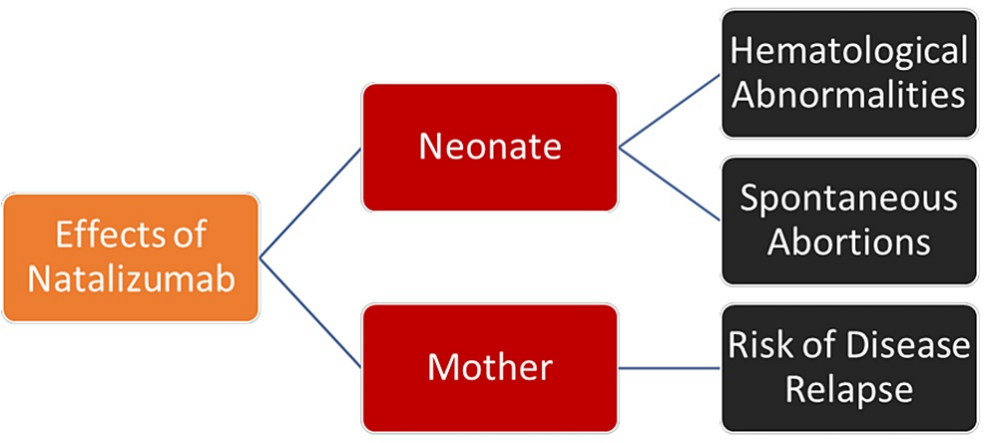
Effects of natalizumab exposure or withdrawal in pregnancy

Figure 2 shows the overall effects of NAT exposure or withdrawal during pregnancy.

Risk of Disease Relapse

A relapse was defined as the onset of one or more symptoms or neurological signs attributable to a central nervous system inflammatory demyelinating event that lasted for at least 24 hours without fever or infection and occurred at a minimum of 30 days after the last relapse [10,11].

In a cohort study conducted in 2020, patients treated with highly effective DMT (H-DMT) were compared to a group of patients treated with moderately effective DMT (M-DMT) and a group with no treatment. NAT and fingolimod were considered a part of the H-DMT. Other immunomodulatory agents were under the M-DMT group. Mean washout periods were significantly higher in the H-DMT group than in the M-DMT group. The results showed that relapses during pregnancy occurred more frequently in the H-DMT group (29%) as compared to the other two groups (<5%). Most of them occurred in the first or the second trimester, within around 13 weeks post-conception and 20 weeks after discontinuation of the H-DMT. Also, the rates of relapse were higher in the first year postpartum in H-DMT patients (53%) than approximately 18% in the other two groups [10].

Although pregnancy had no association with the risk of relapse, it was found that the longer the duration of the washout period, the higher the risk of relapse and disease progression in H-DMT patients. This was not the case in patients receiving M-DMT or no treatment [10].

Similarly, a cohort study conducted in 2018 showed relapses in 36.5% of the patients who received NAT. This is 3.5 folds greater than that observed in women given other immunomodulatory agents or no treatment. This clearly shows that pregnancy is not protective against disease relapse in patients taking NAT before conception [11].

Based on these two studies, it can be inferred that NAT has a significantly high risk of relapse when its usage is suspended compared to other DMTs. As concluded by Portaccio et al., the best way to reduce relapse risk is by avoiding NAT washout and resuming DMT soon after delivery [11].

Hematological Anomalies in Neonates

Although self-resolving anemia is physiologically seen in most newborns at two to three months of life, it is important to give special attention to the clinical onset of anemia related to drugs like NAT [6].

In a study conducted in 2020, out of seven infants exposed to NAT either through pregnancy or breast milk, two had developed mild/moderate hematological abnormalities during breastfeeding, and five had mild to moderate anemia and/or mild thrombocytopenia at birth. It was very reassuring when these abnormal laboratory values were resolved independently, despite a small amount of continuous NAT exposure through breast milk [12]. Some patients have both anemia and thrombocytopenia, while others have neither. These hematologic alterations seem to be associated with receiving NAT treatment, especially during the third trimester of pregnancy [3].

In a case series conducted back in 2014, 10 out of 13 newborns had shown hematological abnormalities, including anemia, thrombocytopenia, and leukocytosis with exposure to NAT during the third trimester of pregnancy. In vitro studies and cynomolgus monkey studies had previously shown that NAT can interfere with hematopoiesis in the fetus. Although one complication of subclinical bleeding was reported, there was no need for a specific treatment for the abnormal counts to resolve [13].

Similarly, a case report from 2021 of a neonate born to a mother who had continued to use NAT during pregnancy showed moderate anemia with a hemoglobin of 10.9 g/dL after birth. All other parameters, including length, weight, head circumference, and other blood cell counts, were within the normal range. Despite iron and folic acid supplementation, subsequent blood samples had shown progressive worsening of the anemia with a hemoglobin level of 8.0 grams per deciliter at 40 days of life. Therefore, erythropoietin (EPO) therapy was started, and monthly blood samples gradually increased blood cell count [6].

Approximately six out of nine articles being reviewed had reported cases of hematological abnormalities in newborns. However, no further complications were noted.

Risk of Spontaneous Abortions

Three studies were reviewed to establish the risk of spontaneous abortions in patients exposed to NAT during or before pregnancy.

A cohort study conducted in 2018 compared three groups from major Italian multiple sclerosis centers: one with exposure to NAT, one with exposure to beta-interferon, and another unexposed. In comparison to the other groups, NAT exposure showed a significantly higher risk of spontaneous abortions (17.4%). However, this rate was not higher than that of the general population [14].

In contrast, another cohort study conducted in 2015 showed no increase in the risk of spontaneous abortions with NAT exposure compared to the disease-matched group [8]. This demonstrates the need for further evaluation.

In addition, a cohort study from 2016 concluded that the rate of spontaneous abortions, although higher when compared to patients in the Registry, is similar to that in the general population [15].

Based on the results of these three studies, it can be concluded that although there is a risk of spontaneous abortions in patients exposed to NAT, the risk is not greater than what is normally observed in the general population. Therefore, spontaneous abortions need not be a determining factor for patients who wish to continue taking NAT during pregnancy.

This systematic review showed that the most common complications of using NAT during pregnancy are spontaneous abortions and mild hematological abnormalities. Even though there is a slight risk of spontaneous abortions, it is not significantly higher than in a normal population. The use of NAT has not aggravated this risk. There is a significant risk of developing hematological abnormalities in the newborn; however, it is mild enough to be resolved with minimal intervention.

Limitations

One major limitation of this systematic review is the availability of data for larger sample sizes. Most of the studies involved small samples, and the data was insufficient to conclude indefinitely. In addition, the lack of information about how physiologic anemia associated with pregnancy may affect the anemia caused by NAT can lead to variability in study outcomes. Moreover, many studies had to be excluded due to not fulfilling the eligibility criteria during the quality check, making them unreliable. This suggests that more studies of better quality need to be conducted and larger sample sizes are required to generalize the outcomes of these data.

## Conclusions

In this systematic review, the main risks of continuing the use of NAT for treating multiple sclerosis during pregnancy were studied. Although NAT is a pregnancy category C drug, a significant risk of disease relapse with its suspension has resulted in the continuation of NAT usage in pregnant patients depending on their disease severity. As per the previous studies, there were some complications in the newborn and the mother; however, since the benefits outweighed the risks, the drug was not discontinued. The most significant adverse outcomes of NAT exposure, especially in the third trimester, were mild anemia and/or thrombocytopenia in the newborn. These laboratory abnormalities were resolved with very minimal intervention in almost all cases. It is advisable for all neonates born to mothers with NAT exposure after conception to be tested for hematological abnormalities to prevent any further complications. Another significant adverse event is the risk of spontaneous abortions. Although quite a few cases of spontaneous abortions have been reported, this risk is almost the same as that of the general population. Therefore, continuous surveillance may be beneficial in reducing the risk.

Overall, NAT can be considered safe for multiple sclerosis treatment during pregnancy; yet, strict monitoring of patients keeping in mind the risk of potential adverse events is required. Better-quality studies involving larger sample sizes need to be conducted to understand further adverse outcomes associated with NAT exposure in pregnancy and how they can be prevented.
